# Supplementary material for: Isolation, validation, and long-term culture of mouse ear fibroblasts
Source: Biol Open. 2026 May 20;15(5):bio062483. doi: 10.1242/bio.062483 (PMC13225709; doi:10.1242/bio.062483)
Supplement: Supplementary information [file biolopen-15-062483-s1.pdf]

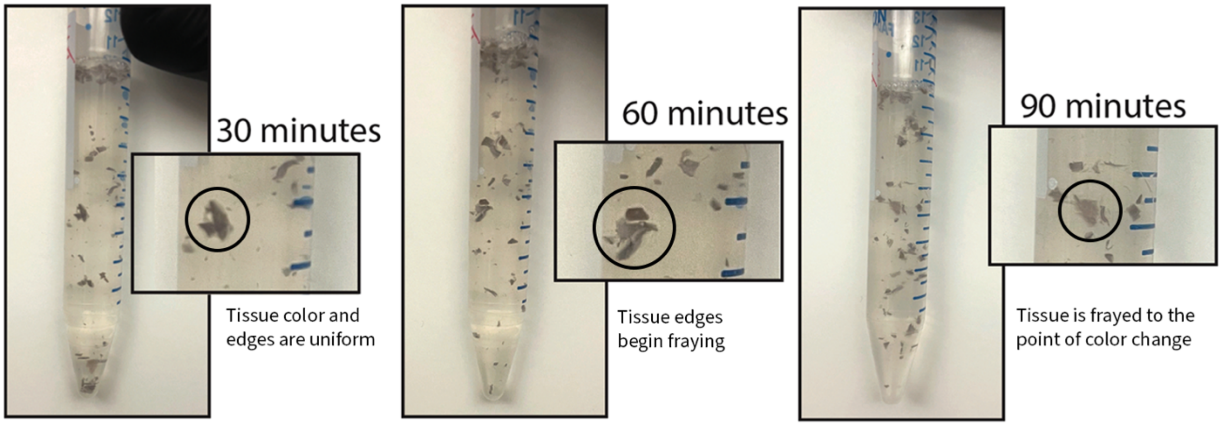

Fig. S1. Visual indicators of mouse ear digestion during Mouse Ear Fibroblast (MEarF) isolation. Representative images of mouse ear tissue digestion in phosphatebu 9ered saline (PBS) containing 1% penicillin-streptomycin (pen/strep) and approximately 14 Wünsch units Liberase TM. Tissue was digested on a rotating platform at 37°C. Images show the progression of digestion at 30, 60, and 90 minutes. Circles highlight representative tissue pieces demonstrating changes in tissue architecture and opacity during digestion. At 30 minutes, tissue edges and color remain intact. By 60 minutes, tissue edges appear frayed. At 90 minutes, optimal digestion is achieved when tissue edges are ex tensively frayed with visible color change, and the solution appears cloudy. Overdigestion results in the complete loss of visible tissue fragments.

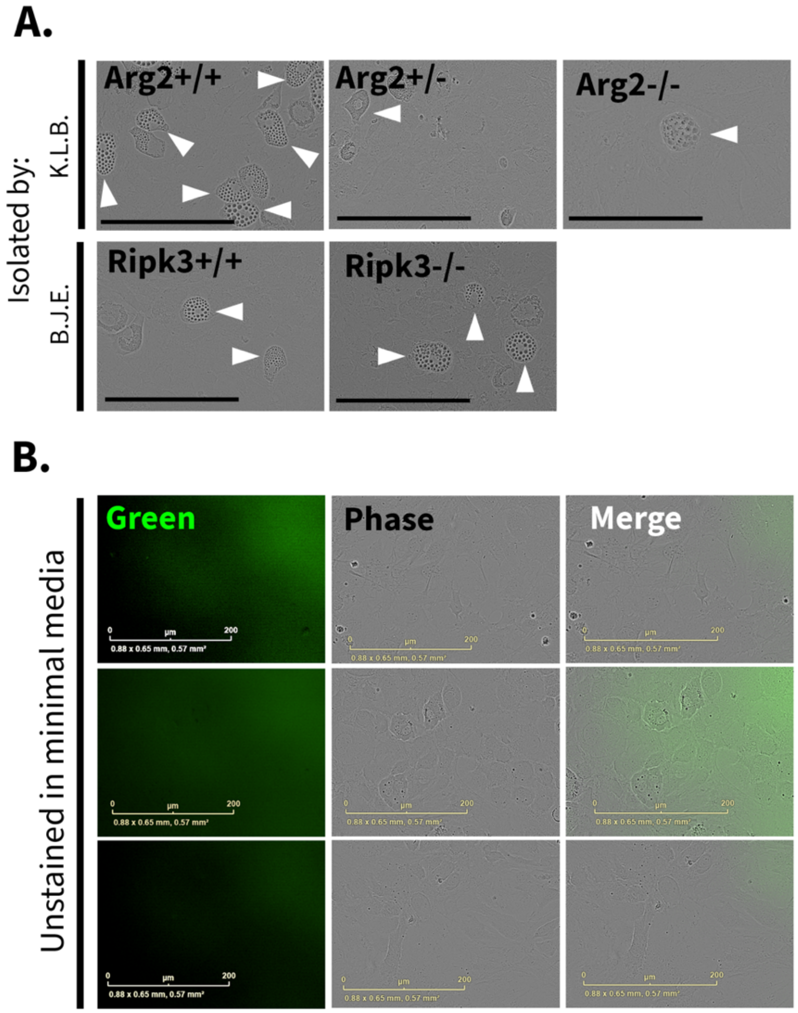

Fig. S2. Minimal autofluorescence in Incucyte green channels and adipocyte-like cells formed in the MEarF cultures regardless of the donor's genotype.

(A) Representative images of MEarFs with adipocyte-like cell contamination, all cultured in enriched media from five different mouse genotypes and two separate researchers' isolation: $Arg2^{+/+}$, $Arg2^{+/-}$, $Arg2^{-/-}$ (Isolated by K.L.B.), $Ripk3^{+/+}$, and $Ripk3^{-/-}$ (Isolated by B.J.E.). Arrowheads indicate examples of adipocyte-like cells observed in MEarF cultures. All images were collected with the Incucyte at 20x magnification, scale bar = 200 μm. Brightness and con trast of images were adjusted linearly and uniformly to enhance visualization. (B) Unstained con trols of mouse ear fibroblasts (MEarFs) cultured in minimal media to demonstrate the in trinsic autofluorescence from the GFP and phase-contrast channels on an Incucyte live cell imaging system with a 20x objective, scale bar = 200 μm, n = 3.

Biology Open • Supplementary information

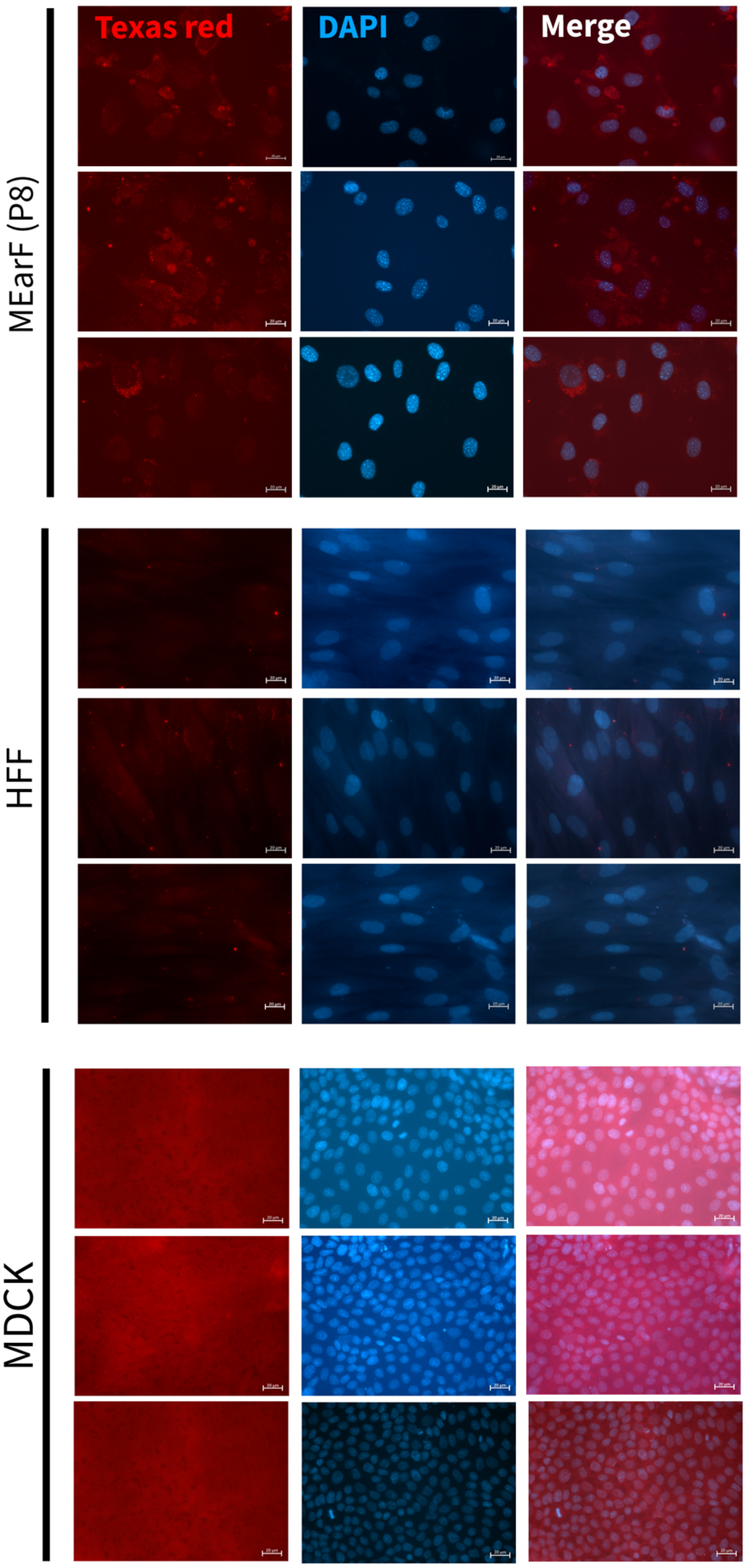

Biology Open • Supplementary information

Fig. S3. Secondary-only controls of human foreskin fibroblasts (HFFs), mouse ear fibroblasts (MEarFs), and Madin-Darby canine kidney cells (MDCKs).

Representative immunofluorescence imaging of passage 8 MEarFs, HFFs, and MDCKs with secondary antibody only (Alexa Fluor 584 (Invitrogen #A-11006) 1:250). Nuclei were counterstained with DAPI (blue). Images were acquired on a Zeiss Axioplan III fluorescence microscope at 40x magnification, scale bar = 200 μm. Brightness and contrast of images were adjusted linearly and uniformly to emphasize the background of the secondary antibody.

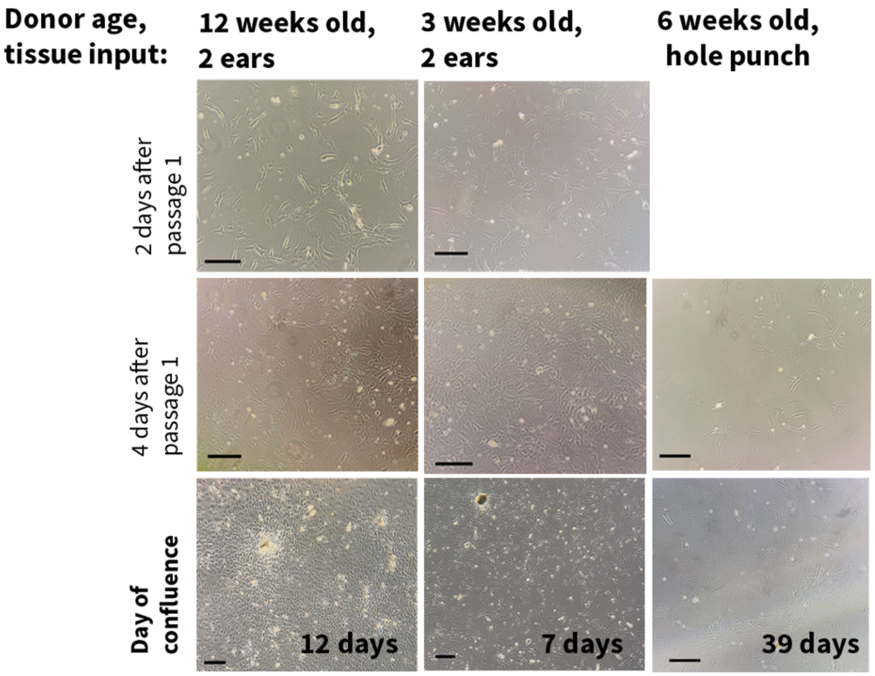

Fig. S4. Relative growth of newly isolated MEarF cells from a range of host tissue donors over passage 0, passage 1, and at confluence.

Mouse donors are from both ears of a 3-week-old, a 12-week-old, and two hole punches of a 6-week-old mouse. The flasks (N=6, n=2) were observed 2 and 4 days after the first passaging, and at the time of confluency. Images were captured through the eyepiece of a 20x objective light microscope with a phone camera. Scale bar = 200 um, n =2. Brightness and contrast of images were adjusted linearly and uniformly to enhance visualization.
